# Supplementary material for: The interactome of intact mitochondria by cross-linking mass spectrometry provides evidence for coexisting respiratory supercomplexes
Source: Mol Cell Proteomics. 2017 Dec 8;17(2):216–32. doi: 10.1074/mcp.RA117.000470 (PMC5795388; doi:10.1074/mcp.RA117.000470)
Supplement: Supplemental Data [file supp_RA117.000470_133922_0_supp_23978_fzffwf.zip › spectra_annotation/mito_spectra_annotation/54-1-13-1-5-1.pdf]

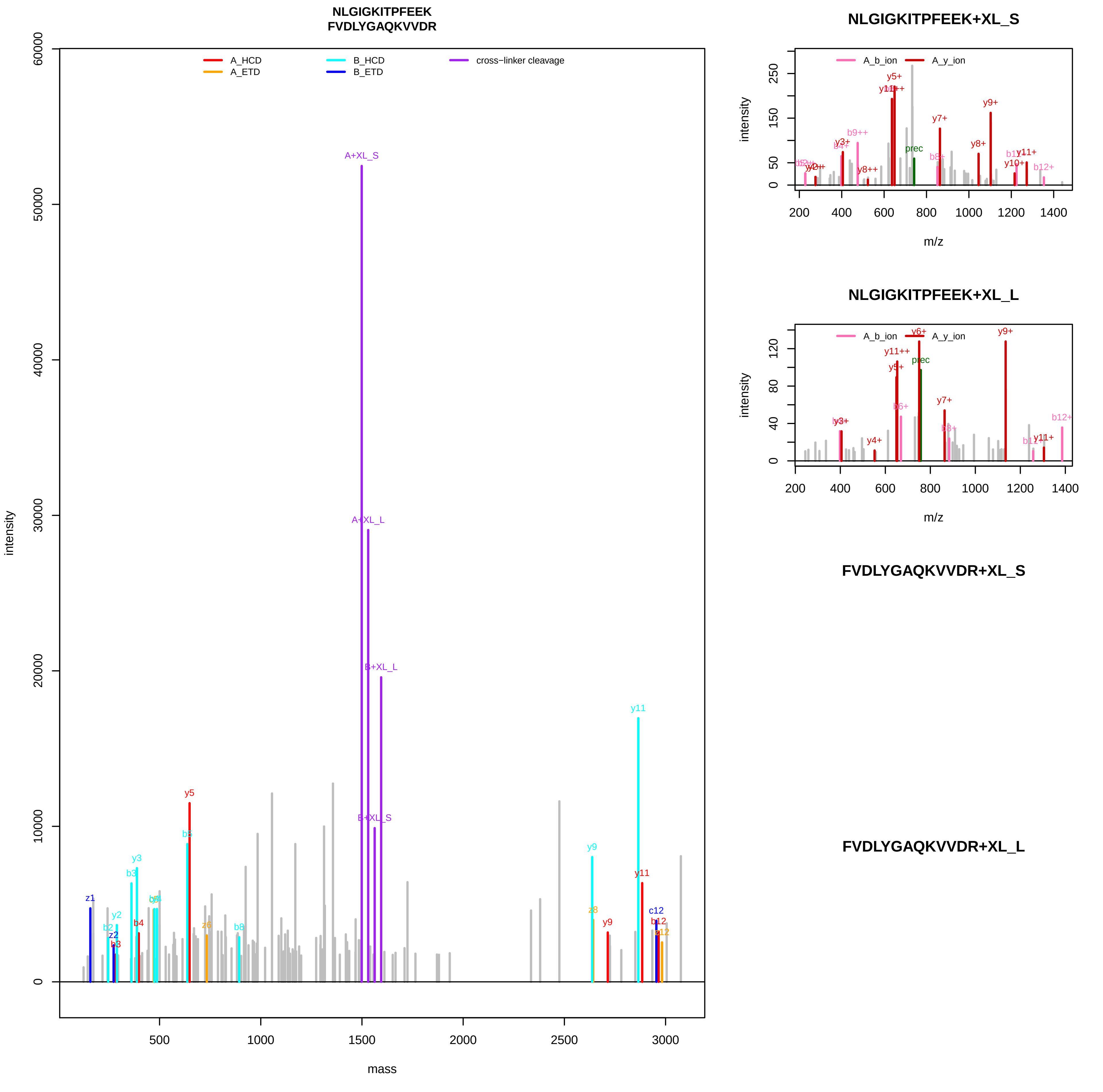
NLGIGKITPFEEK
FVDLYGAQKVVDR
A_HCD
A_ETD
B_HCD
B_ETD
cross−linker cleavage
intensity
mass
A+XL_S
A+XL_L
B+XL_L
B+XL_S
y11
y5
b5
y9
z8
y9
y11
c12
b12
z12
z1
b2
z2
b3
y2
b3
y3
b4
z6
b8
NLGIGKITPFEEK+XL_S
A_b_ion
A_y_ion
intensity
m/z
y5+
y9+
y7+
y8+
y3+
b9++
prec
y8++
y10+
y11+
b12+
NLGIGKITPFEEK+XL_L
A_b_ion
A_y_ion
intensity
m/z
y6+
y9+
y11++
prec
y5+
y7+
b6+
y3+
y4+
y11+
b12+
FVDLYGAQKVVDR+XL_S
FVDLYGAQKVVDR+XL_L
